# An optimal selection method for exterior design schemes of subway trains based on multi-level gray relational analysis

**DOI:** 10.1038/s41598-023-32772-5

**Published:** 2023-04-12

**Authors:** Rui Zou, Ze-Rui Xiang, Jin-Yi Zhi, Tian Li, Hong-Tao Chen, Tie-Cheng Ding

**Affiliations:** 1 Department of Industrial Design, School of Design, Southwest Jiaotong University, Chengdu, China; 2 Institute of Design and Research for Man-Machine-Environment Engineering System, Southwest Jiaotong University, Chengdu, China; 3 State Key Laboratory of Traction Power, Southwest Jiaotong University, Chengdu, China

**Keywords:** Engineering, Mechanical engineering

## Abstract

To make appropriate decisions in the evaluation phase of the exterior design of subway trains, an optimal selection method was proposed based on multi-level gray relational analysis. The exterior design factors of subway trains were analyzed to construct an index system for design evaluation. The significance of each index was compared through an analytic hierarchy process. The correlation coefficient of each index in the plan was calculated through gray relational analysis to obtain the weighted correlation degree of each design scheme. The optimal selection of the exterior design of Guangzhou Metro Line 6 in China was considered as an example. Four types of subjects were recruited: professional designers, students majoring in design, subway train design experts, and subway passengers in Guangzhou. The weight of each index in the evaluation system was calculated using questionnaire scoring. Virtual simulation software was applied to evaluate the human factors related to each scheme. The indices in each plan were then scored to calculate the correlation coefficient and the overall correlation degree; and finally, the optimal selection was obtained. The results showed that it was practical to evaluate and optimize the exterior design of subway trains based on multi-level gray relational analysis. In the evaluation index system, the weights of technology, human factors, aesthetics, and culture were 0.517, 0.297, 0.099, and 0.087, respectively, which showed that technology had the greatest impact on the system, while human factors, aesthetics, and culture were useful complements. Our results showed that Design Scheme 1 was unsuitable as an optimization scheme due to the high escape window. Meanwhile, Design Scheme 2 was optimal overall, from a technical perspective. Design Scheme 3 was the best in terms of the escape window index (a human factor). Design Schemes 3 and 4 were optimally assessed from aesthetic and cultural perspectives. This study is conducive to the optimization of the exterior design of subway trains, can be used to inform design iteration, and provides a reference for the optimal selection of design schemes for other urban rail trains.

## Introduction

An underground railroad, also known as a subway, is an urban rail transit system. An underground railroad has the advantages of a large carrying capacity, high efficiency, convenient riding, and small land occupation^1,2^. Since the completion of the Beijing subway in 1969, underground railways have been greatly developed in China. As of December 31, 2021, a total of 9192.62 km of urban rail transit lines have been put into use in mainland China. Among them, 7253.73 km are subway lines, accounting for 78.9% of the total^3^. With the rapid development of rail traffic infrastructure, subways have become an important means of transportation for travelling in cities^4^. As the core component of the subway train system, it is necessary to conduct relevant research on metro trains.

The industrial design of metro trains refers to the study of all objects and environments related to metro train riders from the perspective of the human–vehicle–environment system, and its research focuses mainly on two aspects: train exterior design and train interior design^2,5^. The design of the exterior of the train mainly includes the exterior shape and paintwork of the train. A good train appearance can meet the aesthetic needs of passengers while meeting technical requirements, thus optimizing the passenger travel experience.

Most of the research on metro train appearance design is proposed from the perspective of design methods. Li et al.^6^ proposed an exterior design approach based on expert and user perspectives, incorporating extension semantics and fuzzy comprehensive evaluation. Meanwhile, Rong et al.^7^ studied the creative design of urban rail vehicle exteriors with the shape grammar method. Qi et al.^8^ proposed an exterior design methodology for subway trains by combining cultural symbols with regional characteristics and bionic images. Liang et al.^9^ proposed a means of optimizing regional cultural factors for the exterior design of urban rail trains. However, there is still a lack of research into the evaluation and selection of subway train design solutions, which is a key step in the aesthetic design process. A rational approach to the evaluation of design solutions can provide a sound basis for the selection of solutions, as well as reveal the right direction for design iterations. Therefore, there is a need for research into the methodology used in the optimization of the exterior design of subway trains.

In the field of industrial design, mathematical models are often used to make preferential decisions on design solutions in order to help researchers make reasonable design decisions. He et al.^10^ proposed an evaluation approach for the design of the train–passenger interface based on the analytic hierarchy process (AHP) and independent weight method; this approach could evaluate test prototypes and existing trains (specifically, of novel double-decker high-speed trains). Li et al.^11^ constructed an optimal decision model for industrial product design schemes based on a multi-level gray comprehensive evaluation method, completed priority ranking of the design schemes of multi-rotor drones, and verified that the results were in accordance with the entropy-weight technique for order of preference by similarity to ideal solution (TOPSIS) method. Tang et al.^12^ set up a fuzzy comprehensive evaluation model of passenger experience, and thus could select the best fabric material used on car seats. By choosing a reasonable mathematical model for the evaluation and selection of design solutions, the design process can avoid decision-making errors caused by the designer’s subjectivity, and make the design solution selection process more objective and rational^13–15^. The AHP can divide complex design problems into levels and determine the importance of each design index, as well as the advantages and disadvantages of each solution, through weight calculation^16^. The grey correlation analysis (GRA) method can quantify the evaluation of design solutions and evaluate the merits of design solutions by comparing the comprehensive scores^17^.

By combining the above two mathematical models, an optimal selection method is proposed based on multi-level gray relational analysis. According to the characteristics of the industrial design of subway trains, a design evaluation index system is constructed. The weights of the design indices are quantified with the AHP, and design schemes are evaluated using GRA. The feasibility of the proposed method is demonstrated by using the optimization of the exterior design scheme of Guangzhou Metro Line 6 in China as an example.

The rest of the paper is organized as follows. Section "Background" provides a description of the research object and the underlying theory of the research method. Section "Optimization of subway train exterior design scheme selection" introduces the optimization of selecting subway train appearance design schemes. Section "Evaluation indices and correlation degrees" constructs the subway train appearance design scheme evaluation index system, and introduces the subway train appearance design scheme selection method in detail. Section "Case study" uses Guangzhou Metro Line 6 in China as an example to verify the selection method. Finally, Section "Conclusions" presents the conclusions.

## Background

### Exterior design of subway trains

In China, subway trains fall into three types^18^: A, B1, and B2. Trains of each type usually consist of two end carriages and several middle carriages. Modeling and painting of the train exterior are the two core research areas of exterior design and are immediately and closely related to the regional culture, operation lines, and passenger aesthetic preferences for the target operating city. The modeled line of the longitudinal symmetric plane and the maximum contour line are the two most essential features that determine the exteriors of trains, and changes are concentrated in the driver’s cab of the end carriage^19^. Therefore, design of the front model is the most significant part of design research. Since the aerodynamic performance requirements for subway trains are not high due to their relatively low speeds, the engine shape design mainly takes into account culture, aesthetics, and human–machine problems inside the driver’s cab, and blunt end shapes with a carriage length within 3000 mm are mostly commonly adopted^2^. The painting of subway trains involves main and auxiliary colors^20^. The main purpose of painting is to promote corrosion resistance and aesthetics of the train body, act as a carrier to publicize regional culture, and perform commercial advertising and marketing^21,22^. Table 1 shows some examples of subway train exteriors with cultural characteristics.

**Table 1:** Some examples of subway train exterior.

Name	Train images	Train shape	Train painting
London subway train		The modeling element originated from the arch of a telephone booth with British characteristics and the front face style of British antique cars	Both the train painting and subway sign refer to the red and blue color of the British flag, featuring regional culture
Moscow subway train		Avant-garde and creative shape. The all-glass design of the train doors provides passengers with maximum open space and security	Decorated with a red ribbon pattern, giving the train a dynamic sense. The overall painting is concise and well-aligned, consistent with the molding style
Singapore subway train		The subway train design was inspired by the vibrant charm of the city. The drum-shaped train body creates a sense of speed	The exterior of the train body adopts red and green pulse curves to highlight the visual effect of passengers’ entry
Chengdu Metro Line 9		The exterior shape of the train body is smooth. The front end is decorated with Light Emitting Diode lamps with dot lights, and the side shape is round and full	The whole train is painted in yellow, the symbol color of Line 9. The sun-god bird pattern manifests the regional culture of Chengdu

### Multi-level gray relational analysis

#### Analytic hierarchy process

The AHP is a multi-criteria decision-making methodology combining qualitative and quantitative analyses, and was presented by Saaty et al.^23^. Based on pairwise comparisons, it breaks relevant elements of a decision problem into an objective, criteria, and schemes to determine the weights of the criteria and the priority of schemes in a structured way^24^.

During the process of decision-making in design, the AHP is a common empowerment method combining the characteristics of quantitative and qualitative analyses. Moreover, it can break down the complex problem of designing layer by layer. The main steps are as follows^25–27^: (1) establish a hierarchy model for the target product, (2) construct a judgment matrix for the comparison, (3) determine the weights, and (4) check the consistency. In the evaluation of the exterior design of subway trains, applying the AHP to the weight analysis of the evaluation criteria can combine a subjective evaluation with an objective analysis to quantify the design evaluation. Therefore, the AHP is considered a simple and effective multi-criteria decision-making tool^28^.

#### Gray relational analysis

GRA, presented by Deng Julong in 1984, is widely used for addressing complex correlations between multiple factors. The basic thought is to estimate the closeness of the connections between sequences^29,30^ in accordance with the level of similarity of different sequence curve geometries.

The adoption of GRA in the calculation of the correlation between the product solution and the ideal solution for multiple indicators can optimize product decisions while obtaining effective product iteration ideas. The main steps are as follows^31,32^: (1) determine the reference and comparison data columns, (2) make the data dimensionless, (3) calculate the gray relational coefficients of the indices, and (4) calculate the gray relational degrees of the schemes. When evaluating subway train exterior design schemes, a correlation calculation is carried out to compare the closeness of the data columns and provide a sound basis for deciding on the preferred design solution.

## Optimization of subway train exterior design scheme selection

**Figure 1:**
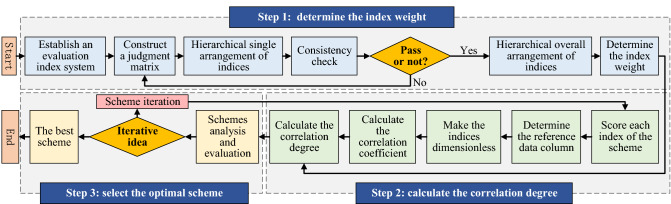
Optimization process of subway train exterior design.

For the exterior design of subway trains, an optimal selection process for the scheme was determined, as shown in Fig. 1. The process includes three steps, as outlined below.

*Step 1:* Determine the index weight. An evaluation index system for the exterior design of subway trains was built from the perspective of industrial design. Indices were compared pairwise to construct a judgment matrix, and index weights were determined through ranking and a consistency check.

*Step 2:* Calculate the correlation degree. Each index in the design scheme was scored, and the highest scores were selected to produce a reference data column. The dispersion degree of the evaluation index in each scheme relative to the reference data column was obtained by calculating the correlation coefficient of the dimensionless data column. Finally, ranking of the advantages and disadvantages of the overall schemes was conducted through calculating the correlation degree of each scheme and combining this degree with the correlation coefficient and weight of each index.

*Step 3:* Select the optimal scheme. In accordance with the correlation coefficient of the index and the final correlation degree, the optimal scheme is selected.

## Evaluation indices and correlation degrees

### Establish an evaluation index system of subway train exterior design

The industrial design of rail trains should focus on the comprehensive consideration of four aspects: technology, aesthetics, human factors, and culture ^2^. By considering these, a design scheme that can meet relevant technical requirements, has a beautiful exterior and pleasant driving environment, and is in accordance with the cultural characteristics of its users can be developed. Therefore, an evaluation index system for train exterior design was built based on these four factors^19^. Details are presented in Table 2.

**Table 2:** Evaluation indices of subway train exterior design.

Target layer	Criterion layer	Index layer	Description
Exterior design evaluation of subway trains S	Technology T	Relevant standards and technical conditions T_1_	In accordance with relevant standards developed by the union of railways, the subway train limit, design basis, basic parameters, and other aspects are explicitly stipulated
		Train body structure T_2_	Requirements of the structural feasibility, economic efficiency, and weight reduction of the train body should be taken into account
		Materials and techniques T_3_	Based on the structural strength and requirements of the train body, the rationality of materials and processing technology, including the processing difficulty, price, strength, maintenance difficulty, and lightweight degree, should be taken into consideration to ensure the best effect of the coordination between various factors
	Human factor H	Escape window H_1_	The size and position of the escape window should fit the driver’s body size and percentile to allow a smooth escape in case of danger
		High/low signal H_2_	The size of the front window should meet the needs of the driver to effectively observe the high and low signals while sitting
	Aesthetics A	Modeling A_1_	The overall exterior and each part of the subway train should be in line with the principle of form beauty, such as the proportions and measurements, balance and stability, unity and change
		Painting A_2_	The color matching of the subway train exterior should be harmonious, conform to the public aesthetic, and be innovative to some extent, which can embody the local cultural characteristics
	Culture C	Material level C_1_	The exterior designs of subway trains should reflect the characteristics of the material aspect of the regional culture, including the natural scenery, architecture relics, animals and plants, clothing, and local specialties
		System level C_2_	The exterior design of subway trains should be in accordance with the local behaviors, systems and regulations, customs and traditions, and ethnic relations
		Spiritual level C_3_	The exterior design of subway trains should embody the mental structure, philosophy, and religious thoughts of the local people

#### Technological factors

The technological factors are the basis of the exterior design of subway trains. The vehicle limit, vehicle marshalling, body structure, signal visibility, and safety escape features of subway trains are stipulated in relevant standards and technical specifications (such as UIC 651, EN 45,545–4, GB 50,157, GB 10,000, GB/T 3091, GB/T 5914.2, GB/T 7928, and CJJ/T 96). These standards and specifications should be strictly followed in the industrial design of subway trains. The material and technology of the train body are related to the structural strength and structural form, which also influence the final design effect. Furthermore, as an urban rail traffic vehicle with a large load, the material selection of subway trains should comprehensively take into consideration the service life, economic efficiency, and easy molding and processing. Since the speeds of existing subway trains in China are mostly within 100 km/h, and thus are not much affected by air resistance and pressure waves of trains passing, pneumatic performance was not considered here^2,33^.

#### Human factors

In terms of human factors, people that should be considered in the industrial design of subway trains include passengers, drivers, maintainers, and other related personnel. The exterior design of subway trains focuses on the exterior of the train front, which mainly involves the train driver. The human factors of the exterior design of subway trains related to the train driver mainly involve two indices: the “escape window,” and the “high/low signal”. “Escape window” refers to the size and position of the emergency escape window accommodating the driver’s size. Meanwhile, “high/low signal” refers to the size of the front window meeting enabling the driver to effectively observe both high and low signals while sitting.

#### Aesthetic factors

Aesthetic factors in the exterior design of subway trains mainly involve the beauty of the train’s shape and painting. Shape and painting should be based on relevant principles of form beauty^34^. Additionally, the painting should be in line with the public aesthetic of the target region.

#### Cultural factors

Cultural factors involved in the exterior design of subway trains include cultural modeling factors, especially historical relic modeling and natural ecological modeling, cultural color factors, natural color, color of historical remains, and cultural connotation factors; the latter should further stress the natural ecology and historic culture^9^.

### Weight calculation of evaluation indices of subway train exterior design

The evaluation index system is divided into a target layer, a criterion layer, and an index layer. After the establishment of the evaluation index system, pairwise comparison of the elements within the system is required to construct the judgment matrix $C$:

1 $$ C = (c_{ij} )_{n \times n} , $$

where $n$ represents the number of indices, $c_{ij}$ is the importance value of factors $i$ and $j$ relative to the target, and $i,\;j = 1,\;2,\; \ldots ,\;n$. Calculating the relative importance of an index to its corresponding criterion of the upper layer in accordance with the judgement matrix is called hierarchical single arrangement. The root method is applied to conduct the hierarchical single arrangement of the judgment matrix. The product $M_{i}$ of the elements in each row is worked out based on the value of the judgment matrix:

2 $$ M_{i} = \prod\limits_{j = 1}^{n} {c_{ij} } . $$

The *n*-th root $\overline{{W_{i} }}$ of $M_{i}$ is calculated as follows:

3 $$ \overline{{W_{i} }} = \sqrt[n]{{M_{i} }}. $$

The vectors are normalized to obtain the relative weight *W*_i_:

4 $$ W_{i} = \frac{{\overline{{W_{i} }} }}{{\sum\limits_{j = 1}^{n} {\overline{{W_{j} }} } }}. $$

To ensure the consistency of each judgment and avoid contradictory results when experts judge the index weight, a consistency check of the constructed judgment matrix is required. The maximum eigenvalues λ_max_ of the judgement matrix are calculated first:

5 $$ \lambda_{max} = \mathop \sum \limits_{i = 1}^{n} \frac{{(AW)_{i} }}{{nW_{i} }}, $$

where $(AW)_{i}$ represents the sum of the products of $W_{i}$ and the value in the *i-*th row of the judgment matrix. Then, the consistency index of the judgment matrix *CI* is calculated as follows:

6 $$ CI = \frac{{\lambda_{max} - n}}{n - 1}. $$

The consistency of the matrix is checked by the ratio of the *CI* value to the *RI* value. *RI* is the random consistency index, and its value is different for judgment matrices of different orders^35^, as shown in Table 3. The ratio of *CI* to *RI* is called the consistency ratio and is denoted as *CR*:

7 $$ CR = \frac{CI}{{RI}}. $$

**Table 3:** Consistency index of the judgement matrix.

Order	1	2	3	4	5	6
*RI* value	0	0	0.52	0.89	1.12	1.26

When $CR < 0.1$, the judgment matrix is considered to have satisfactory consistency; otherwise, it will require adjustment.

After completing the hierarchical single arrangement and consistency check of the judgment matrix, hierarchical overall arrangement is required. This allows us to rank the relative importance of the lowest factor (index layer) relative to the highest (target layer). The weight of the index layer relative to the target layer is the product of the relative weights at each layer.

### Correlation degree calculation of subway train exterior design

A reference data column needs to be specified in GRA^29^. The reference data column is an ideal standard of comparison, often consisting of either the optimal value or worst value of each index. In this paper, the optimal value of each index is selected to form the reference data and is denotedf as *x*_0_:

8 $$ x_{0} = \left\{ {x_{0} \left( 1 \right),x_{0} \left( 2 \right), \cdots ,x_{0} \left( n \right)} \right\}. $$

Suppose the number of schemes for comparison is *m*, and the comparison data column in the correlation degree analysis is denoted as $x_{k}$. Then,

9 $$ x_{k} = \left\{ {x_{k} \left( 1 \right),x_{k} \left( 2 \right), \cdots ,x_{k} \left( n \right)} \right\}, $$

where *k* = 1, 2, …, *m*.

When the dimensions of all indices are the same, there is no need to make the indices dimensionless. Thus, the absolute difference $\left| {x_{0} \left( i \right) - x_{k} \left( i \right)} \right|$ of the data column is then calculated, and the maximum and minimum absolute difference values (${\Delta }max$ and ${\Delta }min$, respectively) are defined as below:

10 $$ \Delta max = \mathop {\max }\limits_{k} \mathop {\max }\limits_{i} \left| {x_{0} \left( i \right) - x_{k} \left( i \right)} \right|, $$

11 $$ \Delta min = \mathop {\min }\limits_{k} \mathop {\min }\limits_{i} \left| {x_{0} \left( i \right) - x_{k} \left( i \right)} \right|. $$

The relative difference between the* i-*th index $x_{k} \left( i \right)$ of the *k*-th scheme and the *i-*th optimal index $x_{0} \left( i \right)$ is the correlation coefficient of $x_{i}$ with respect to $x_{0}$ at time *k*. This is denoted as $\xi_{k} \left( i \right)$, and is expressed as

12 $$ \xi_{k} \left( i \right) = \frac{\Delta min + \rho \Delta max}{{\left| {x_{0} \left( i \right) - x_{k} \left( i \right)} \right| + \rho \Delta max}}, $$

where $\rho$ is the resolution ratio. $\rho \in [0,\;1]$, and, generally, $\rho = 0.5$.

The correlation coefficient indicates the correlation degree between each index. An evaluation system contains multiple evaluation indices. To comprehensively assess design scheme *k*, the correlation coefficient needs to be integrated. The integrated value is named the correlation degree and is denoted as *r*_k_:

13 $$ r_{k} = \mathop \sum \limits_{i = 1}^{n} W_{i} \times \xi_{i} \left( k \right). $$

## Case study

### Design samples

**Figure 2:**
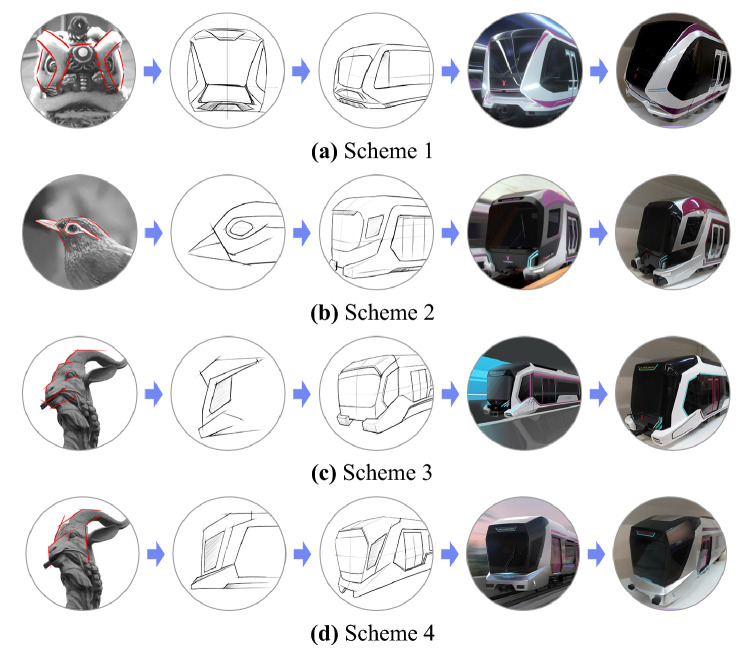
Exterior design scheme of Guangzhou Subway train Line 6, China.

Figure 2 shows four exterior design schemes of subway trains incorporating the elements of regional culture in Guangzhou, China. The technical requirements of Guangzhou Metro Line 6, signal and emergency escape regulations, the principle of form beauty, and cultural constraints of urban regions were fully considered. Design Scheme 1, taking the lion dance of Guangzhou folk culture as the design element, integrates modeling elements of the lion into the front design of the subway train after abstract extraction, thereby presenting a dignified overall model and implying good luck and happiness. Design Scheme 2 takes Garrulax canorus, the city bird of Guangzhou, as the design element by extracting its typical white eyebrow for painting on the train body. The application of this abstraction gives the subway train a sense of modernity. Design Scheme 3, using a sculpture of Wuyang (Guangzhou of China is also call “Yangcheng,” meaning the city of goats) and Guangzhou Tower as painting inspiration, adds modernity to the train’s exterior while maintaining regional cultural characteristics. Lastly, Design Scheme 4, taking a Wuyang sculpture as the design element, abstracts head lines to the modeling and painting, produces an aesthetic image of a goat.

### Optimization and decision-making process

The optimal of the four design schemes was determined by asking test subjects to fill in questionnaires and by conducting virtual simulations. We also conducted an index weight comparison, and index scoring of the design schemes. Index weights were obtained from evaluations made by professional subway train designers (10) and students majoring in design (38). People participating in the index scoring of the design schemes included professional designers (10), students with design majors (38), train body design experts (30), and subway passengers in Guangzhou, China (317). Table 4 shows the personnel classification and scoring tasks. Due to the difficulty of evaluating the human factors by directly viewing a train design sketch, virtual simulation software was adopted to conduct simulation evaluations^36–38^. JACK™ was selected to create the digital human model to assess the escape window and the high/low signals indices for all four design schemes. The upper and lower limit values of the digital human model were determined in accordance with relevant standards or specifications. By simulating driver operations, experts graded deviations between indices and relevant standards or specifications.

**Table 4:** Classification and tasks of index scorers.

Personnel classification	Brief introduction	Qty	Task of index scoring
Professional designers	Product designers with rich experience in exterior design of subway trains	10	Technical, human factor, aesthetic, and cultural indices
Students in design major	Students with design majors who have knowledge of subway train exterior design and practical experience of design	38	Technical, human factor, aesthetic, and cultural indices
Experts of subway train body design	Experts of subway train body design who know the structures of subway train bodies and related technical standards	30	Technical and human factor indices
Subway passengers	Residents who often take the subway and know about the regional culture of Guangzhou	317	Aesthetic and cultural indices

#### Virtual simulation evaluation

In Rhino™, a 1:1 three-dimensional model of the driver’s cab was constructed for the four design schemes. Following the regulations on the selection of railway drivers in China, the 95th percentile of adult Chinese male height (P95) was selected as the upper limit of the driver’s height, while the 10th percentile of adult Chinese male height (P10) was selected as the lower limit of the driver’s height (note that the majority of Chinese drivers are male and the height of P10 males is 160.4 cm^39^, which is close to the provisions in TB/T 3091^40^). Table 5 shows a sample analysis chart of the simulation of the emergency escape window and a simulation of high/low signal visibility for each of the four design schemes. The following can be concluded from the analysis.

**Table 5:**
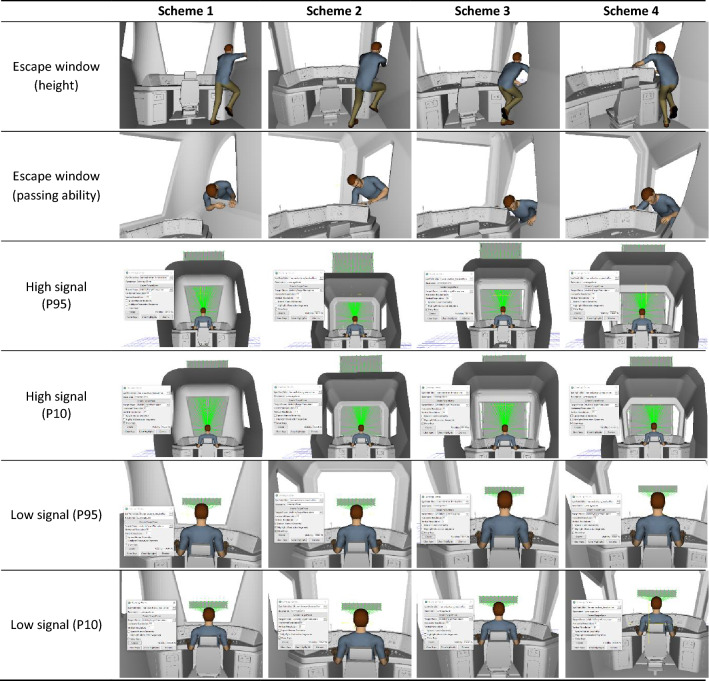
Sample analysis chart of virtual simulation of the design schemes.

(1) The escape window dimensions in all four design schemes meet the space needs for the P95 Chinese male, according to virtual simulations. Thus, the schemes comply with the requirements of EN 45,545–4^41^. However, since the window in Design Scheme 1 is too high for the P10 Chinese male to climb up to and escape, this scheme is not optimal.

(2) When a driver assumes a sitting position, the high signal is mainly blocked by the left, right, and top parts of the front window, while the low signal is mainly blocked by the left, right, and bottom parts of the front window. In accordance with GB/T 5914.2, both high and low signals should be unobscured^42^. Using JACK™, we analyzed the high/low signal visibility for the P10 and P95 Chinese males in a seated posture. The results showed that in all four design schemes the high/low signals were 100% visible and thus met requirements.

#### Questionnaire survey

The questionnaire survey consisted of two phases: weight comparison of evaluation indices, and determining the correlation degrees of the design schemes. In the weight comparison phase, professional designers and students with design majors conducted pairwise comparisons of the importance of each evaluation index by filling out a paper questionnaire (Appendix A) according to the evaluation index system shown in Table 2. To quantify comparisons, a 1–9 scale was adopted.

In the second phase, after viewing three exterior design schemes of subway trains (Design Scheme 1 was no longer an option due to its poor simulation analysis results in terms of the escape window), participants scored each scheme on the tasks in Table 4. The score range was 0–100 points, with five intervals: “failed (0–59 points),” “barely passed (60–69 points),” “average (70–79 points),” “good (80–89 points),” and “excellent (90–100 points).” An in-person questionnaire was given to professional designers and students with design majors (Appendix B), and the design schemes were explained by the interviewer. However, due to restrictions imposed by geographic location and COVID-19, the in-person questionnaire could not be administered for the train body design experts and subway passengers; for these participants, the survey was carried out on WJX™ (Appendix C).

This study was approved by the Ethics Committee of Southwest Jiaotong University and conducted according to the principles of the Declaration of Helsinki. All participants provided informed consent before participating.

### Data processing

#### Index weight

The judgment matrix of the criterion layer of the subway train exterior design evaluation index was obtained by calculating the mean value of the evaluation data obtained from 48 subjects, as shown in Table 6. The largest eigenvalue $\lambda_{max} = 4.07$ of the judgment matrix was produced based on Eqs. (2)–(5), and $CI = 0.02$ was determined according to Eq. (6). The *RI* value of the level 4 matrix obtained from Table 3 was 0.89, and the *CR* value of the judgment matrix worked out from Eq. (7) was.

**Table 6:** Judgment matrix of criterion layer factors.

	Technology	Human factor	Aesthetics	Culture
Technology	1.00	2.45	4.56	4.70
Human factor	0.41	1.00	3.72	3.83
Aesthetics	0.22	0.27	1.00	1.26
Culture	0.21	0.26	0.79	1.00

$$CR = \frac{CI}{{RI}} = 0.02 < 0.1$$.

Thus, the judgment matrix of the criterion layer passed the consistency test.

The weights of the four factors of the criterion layer relative to the target layer were obtained according to Eq. (3). Next, the weights of the 10 factors in the index layer were calculated successively, as shown in Table 7.

**Table 7:** Weights of the evaluation index of subway train exterior design.

Target layer	Criterion layer	Weight	Index layer	Weight
Evaluation of subway train exterior design S	Technology T	0.517	Relevant standards and technical conditions T_1_	0.591
			Train body structure T_2_	0.271
			Materials and techniques T_3_	0.139
	Human factor H	0.297	Escape window H_1_	0.702
			High/low signal H_2_	0.299
	Aesthetics A	0.099	Modeling A_1_	0.700
			Painting A_2_	0.300
	Culture C	0.087	Material level C_1_	0.503
			System level C_2_	0.326
			Spiritual level C_3_	0.171

#### Design scheme correlation degree

The α values for Design Schemes 2–4 were 0.887, 0.900, and 0.889, respectively, suggesting a high reliability of the questionnaire. Kaiser–Meyer–Olkin (KMO) and Bartlett test results were obtained through principal component analysis of the data. The KMO values of the questionnaires of Schemes 2–4 were 0.842, 0.737, and 0.847, respectively. Since these values were larger than 0.6, they met the requirements of principal component analysis. This indicated that the data could be used for subsequent analysis. Furthermore, the data passed Bartlett’s sphericity test (with a significance of less than 0.05), suggesting fitness for factor analysis. The scores of each factor of the questionnaire were analyzed using principal component analysis to extract five common factors. The sum of squares of the accumulated rotating load was 75.293%, showing a high validity of the questionnaire.

By processing the scores produced by the four types of subjects, the initial evaluation data of the three exterior design schemes were obtained (see Table 8). Since the simulated high/low signals in the three design schemes were all 100% visible, a unified value of 85.00 was taken in accordance with the evaluation scale in Table 6. Following Eq. (8), the optimal value of each index was obtained to construct the reference data column:

$$x_{0} = \left\{ {{100,}\;{100,}\;{100,}\;{100,}\;{100,}\;{100,}\;{100,}\;{100,}\;{100,}\;{100}} \right\}$$.

**Table 8:** Scheme initial evaluation data.

Index	Professional designers			Students in design major			Experts of subway train body design			Subway passengers		
	Scheme 2	Scheme 3	Scheme 4	Scheme 2	Scheme 3	Scheme 4	Scheme 2	Scheme 3	Scheme 4	Scheme 2	Scheme 3	Scheme 4
T_1_	68.300	70.600	66.800	81.526	82.447	81.053	85.533	79.400	77.433	/	/	/
T_2_	72.700	71.700	68.000	82.895	82.763	83.026	86.633	79.900	78.933	/	/	/
T_3_	73.800	73.900	71.800	82.053	84.263	84.474	85.300	79.667	77.333	/	/	/
H_1_	67.400	82.900	73.600	70.342	87.289	78.737	74.233	78.667	75.267	/	/	/
H_2_	85.000	85.000	85.000	85.000	85.000	85.000	85.000	85.000	85.000	/	/	/
A_1_	79.700	80.800	76.600	83.289	83.447	82.842	/	/	/	72.246	72.533	72.032
A_2_	71.900	83.400	75.500	83.026	85.737	80.053	/	/	/	70.596	71.032	71.142
C_1_	71.400	78.000	74.600	84.184	82.579	80.711	/	/	/	70.921	71.669	72.227
C_2_	73.000	76.200	74.400	81.500	82.211	82.053	/	/	/	70.041	71.416	72.013
C_3_	71.100	76.500	77.900	82.368	82.605	81.553	/	/	/	71.098	72.587	73.940

The absolute difference between the real scheme data column and the reference data column was calculated, and the maximum and minimum values were determined via Eqs. (10) and (11) to be $\Delta max = 33.200$ and $\Delta min = 12.711$, respectively. When $\rho$ was 0.5, the correlation coefficient of each index in each scheme could be determined according to Eq. (12), as shown in Table 9.

**Table 9:** Correlation coefficients of scheme indices.

Index	Professional designers			Students in design major			Experts of subway train body design			Subway passengers		
	Scheme 2	Scheme 3	Scheme 4	Scheme 2	Scheme 3	Scheme 4	Scheme 2	Scheme 3	Scheme 4	Scheme 2	Scheme 3	Scheme 4
T_1_	0.607	0.637	0.589	0.836	0.858	0.825	0.943	0.788	0.748	/	/	/
T_2_	0.668	0.653	0.603	0.870	0.866	0.873	0.978	0.799	0.778	/	/	/
T_3_	0.685	0.686	0.654	0.848	0.906	0.912	0.936	0.794	0.746	/	/	/
H_1_	0.596	0.870	0.682	0.634	1.000	0.774	0.692	0.773	0.709	/	/	/
H_2_	0.928	0.928	0.928	0.928	0.928	0.928	0.928	0.928	0.928	/	/	/
A_1_	0.794	0.819	0.733	0.880	0.884	0.868	/	/	/	0.661	0.665	0.658
A_2_	0.656	0.883	0.713	0.873	0.950	0.802	/	/	/	0.637	0.643	0.645
C_1_	0.648	0.759	0.698	0.904	0.862	0.817	/	/	/	0.642	0.652	0.661
C_2_	0.672	0.726	0.695	0.835	0.852	0.848	/	/	/	0.630	0.649	0.657
C_3_	0.644	0.731	0.757	0.856	0.862	0.836	/	/	/	0.644	0.666	0.687

**Figure 3:**
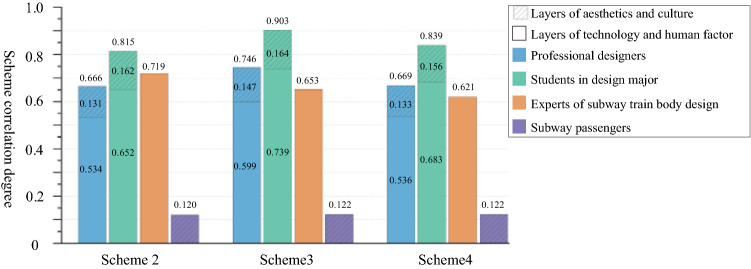
Correlation degrees and rankings of each scheme.

Next, the weighted correlation degrees of the three design schemes were calculated. The larger the correlation degree value, the better the entire scheme. The train body design experts and passengers only scored some of the criterion layer indices. Therefore, the data provided by the design experts only considered the index correlation degree of the technical layer and the human factor layer, while the data provided by passengers only considered that of the aesthetic layer and the culture layer. The weighted correlation degrees of each design scheme were plotted in Fig. 3.

### Results and discussion

#### Comparison of index weights

It can be concluded from Table 7 that the weights of the technology, human factor, aesthetics, and culture indices were 0.517, 0.297, 0.099, and 0.087, respectively. Thus, technology was the basis of the system, while human factors, aesthetics, and culture were beneficial complements to the design. The weight ranking of the four factors was as follows: technology > human factor > aesthetics > culture. Therefore, in the exterior design of subway trains, designers should, while giving due recognition to technical constraints, meet the requirements of drivers’ vision and escape in their designs. Furthermore, integration of aesthetic and cultural factors in the design should not be neglected.

#### Comparison of optimal design scheme selection

From Tables 8 and 9, it can be seen that the professional designers believed that the index scores of the standards and conditions (T_1_), escape window (H_1_), material level (C_1_), and spiritual level (C_3_) of Design Scheme 2 were low, indicating that Design Scheme 2 was inadequate in terms of these four indices. Design Scheme 3 was inadequate in terms of index T_1_. Design Scheme 4 was inadequate in terms of indices T_1_ and train body structure (T_2_). Students majoring in design argued that Design Scheme 2 was inadequate in terms of H_1_. There were no evident drawbacks in Design Scheme 3, but it could be further optimized in terms of technology and culture indices. Design Scheme 4 was slightly inadequate in terms of H_1_. Train body design experts claimed that Design Scheme 2 was inadequate in terms of H_1_. While there were no evident drawbacks in Design Scheme 3 in terms of technology and human factors, Design Scheme 2 was better in terms of the technology index. Furthermore, Design Scheme 4 was inadequate in terms of H_1_, and further optimization in its technology index was required. The index scores of the aesthetics and culture layers given by subway passengers were basically the same across all design schemes, and were lower than those given by professional designers and students with design majors. However, the average score of each index was above 70 points, suggesting that the passengers were basically satisfied with all three design schemes in terms of the aesthetics and culture layers; therefore, the passengers decided that there were no significant design defects in the three design schemes.

When ranking the three design schemes comprehensively, professional designers held that Design Scheme 3 > Design Scheme 4 > Design Scheme 2, and there were no significant differences in the scores of the three schemes at the technology level. Design Scheme 3 was the best in terms of H_1_, aesthetics, and culture. Students with design majors asserted that Design Scheme 3 > Design Scheme 4 > Design Scheme 2, and there were no significant differences in the scores of the three schemes in terms of T_1_, T_2_, modeling (A_1_), and culture. Design Scheme 3 was optimal in terms of the remaining indices. Train body design experts decided that Design Scheme 2 > Design Scheme 3 > Design Scheme 4. Design Scheme 3 was the best in terms of H_1_, while Design Scheme 2 was best in terms of the technology index. Thus, Design Scheme 2 obtained the highest overall score. Subway passengers had relatively consistent acceptance of the three schemes, and the overall scores of Schemes 3 and 4 were slightly higher than that of Design Scheme 2.

Therefore, Design Scheme 2 was optimal overall from a technology perspective. From the human factors perspective, Design Scheme 3 was optimal. Schemes 3 and 4 were optimal from the aesthetic and cultural perspectives. Designers should, when designing the exteriors of subway trains, take various design indices into consideration so that their design schemes conform to all the indices involved in technology, human factors, aesthetics, and culture. Ignoring any one of these indices may result in rejection of the scheme due to its limitations.

#### Differences in index scorers

When evaluating design schemes, it was found that the average score of professional designers was significantly lower than that of the student group, and the score gap was the most significant when it came to the technology index. With relevant professional knowledge, students with design majors could provide sound advice for the optimal selection of subway train design schemes to a certain degree. However, since they lacked practical experience in design, rationality and feasibility were not taken into consideration in the design process and evaluation. In contrast, professional designers with more rich experience in design could take into account various aspects of design indices in a more comprehensive way when evaluating design schemes. Moreover, they would focus on technology factors in the design and design evaluation process by paying more attention to the rationality and feasibility of the design schemes. Compared with professional designers, train body design experts could observe more technical details and defects in the design schemes. Therefore, before designing the exteriors of subway trains, professional designers should cooperate with train body design experts to determine design boundaries to avoid technical defects in the design scheme, thus reducing modification costs required by design iteration. Furthermore, techniques used in creating design schemes should be verified based on the advice of train body design experts during design optimization. Furthermore, designers should gather opinions of local subway passengers widely in the early stages of design so that exteriors are consistent with the public aesthetic with full reflection of regional cultural characteristics.

Design is a typical ill-structured problem, for which there is neither a clear problem solution method nor a unique solution, and there is a problem of delayed feedback^43^. A good design requires problems to be constructed by defining product characteristics, and products must be evaluated by establishing personalized systems. By analyzing the main design factors involved in the exterior design of subway trains, this paper provides some specific indices, from the aspects of technology, human factors, aesthetics, and culture that can provide a reference for urban rail subway train designs.

## Conclusions

High-quality exterior design of subway trains can not only provide an enjoyable experience for passengers when traveling but can also contribute to the promotion of the cultural image of a city. To assist designers in making sound decisions in the scheme evaluation phase, this paper proposes an optimal selection method for exterior design schemes of subway trains based on multi-level GRA. Construction of an evaluation index system on the basis of the AHP can break down complex design problems layer by layer. Furthermore, calculating the correlation degrees of the schemes with GRA can provide effective iterations of ideas to realize optimization decisions in the design schemes. Illustrated by a design case, the optimal selection of design schemes was performed using questionnaires and virtual simulations. We found that the evaluation method based on multi-level GRA provides reference iteration direction for designers, along with theoretical support for final scheme decisions. The evaluation method is feasible and effective.

Through conducting passenger questionnaires, it was discovered that there was a great difference between the scores given across passengers due to the influence of their living environment, education, and lifestyle. However, since the group of passengers was not subdivided, when the number of subjects was large, the results of the processed questionnaires could not reflect the differences between these various types of passengers. Hence, our research methodologies could be further improved to classify the groups more finely. In addition, when evaluating the indices of technology and human factors by viewing images of the design schemes, the subjects often discovered distinct technical defects without seeing more detailed differences between the design parameters of each scheme. Therefore, further study is required to determine how to evaluate the technology indices of the design schemes more scientifically and intuitively.

## Figures and Tables

**Table 12:**
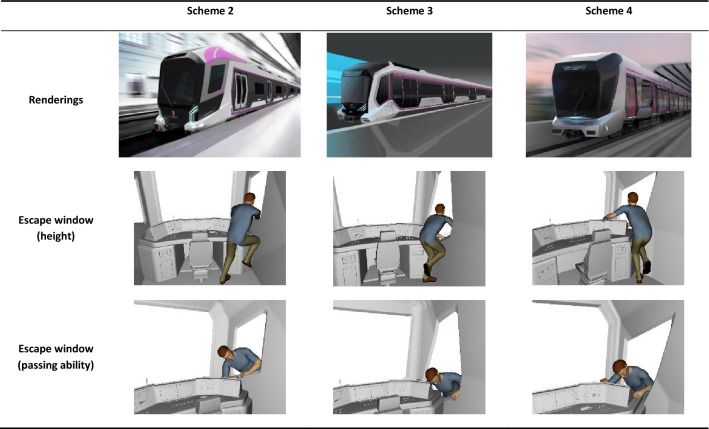
Renderings of the design schemes and escape window virtual simulation analysis chart.

## Data Availability

The data that support the findings of this study are available on request from the corresponding author. The data are not publicly available due to privacy or ethical restrictions.

## Appendixes

### A. Questionnaire of evaluation index weight comparison

Table 10 shows the evaluation indices of the subway train exterior design, including 10 indices of four criterion layer factors. Please read the details to understand the meaning of each evaluation index.

**Table 10:** Evaluation indices of subway train exterior design.

Target layer	Criterion layer	Index layer	Description
Exterior design evaluation of subway trains S	Technology T	Relevant standards and technical conditions T_1_	In accordance with relevant standards developed by the union of railways, the subway train limit, design basis, basic parameters, and other aspects are explicitly stipulated
		Train body structure T_2_	Requirements of structural feasibility, economic efficiency, and weight reduction of the train body should be taken into account
		Materials and techniques T_3_	Based on the structural strength and requirements of the train body, the rationality of materials and processing technology, including the processing difficulty, price, strength, maintenance difficulty, and lightweight degree, should be taken into consideration to ensure the best effect of the coordination between various factors
	Human factor H	Escape window H_1_	The size and position of the escape window should fit the driver’s body size and percentile to allow smooth escape in case of emergency
		High/low signal H_2_	The size of the front window should meet the needs of the driver to effectively observe the high and low signals while sitting
	Aesthetics A	Modeling A_1_	The overall exterior and each part of the subway train should be in line with the principle of form beauty, such as the proportions and measurements, balance and stability, unity and change
		Painting A_2_	The color matching of the subway train exterior should be harmonious, conform to the public aesthetic, and be innovative to some extent, which can embody the local cultural characteristics
	Culture C	Material level C_1_	The exterior design of subway trains should reflect the characteristics of the material aspect of regional culture, including the natural scenery, architectural relics, animals and plants, clothing, and local specialties
		System level C_2_	The exterior design of subway trains should be in accordance with the local behaviors, systems and regulations, customs and traditions, and ethnic relations
		Spiritual level C_3_	The exterior design of subway trains should embody the mental structure, philosophy, and religious thoughts of the local people

Table 11 shows the interpretation of the assignments when comparing the significance of the indices, in which $i$ is the vertical index and $j$ is the horizontal index.

**Table 11:** Index evaluation scale and its meaning.

Order number	Importance level	Assignment
1	Factors *i* and are equally important	1
2	Factor *i* is slightly more important than factor *j*	3
3	Factor *i* is significantly more important than factor *j*	5
4	Factor *i* is significantly more important than factor *j*	7
5	Factor *i* is extremely more important than factor *j*	9
6	Factor *i* is slightly less important than factor *j*	1/3
7	Factor *i* is significantly less important than factor *j*	1/5
8	Factor *i* is significantly less important than factor *j*	1/7
9	Factor *i* is extremely less important than factor *j*	1/9

Please compare the significance of each evaluation index in the following table pairwise in accordance with the above evaluation index descriptions and scales.

**Table:**

	Technology	Human factor	Aesthetics	Culture
Technology	1			
Human factor	\	1		
Aesthetics	\	\	1	
Culture	\	\	\	1

**Table:**

	Relevant standards and technical conditions	Train body structure	Materials and techniques
Relevant standards and technical conditions	1		
Train body structure	\	1	
Materials and techniques	\	\	1

**Table:**

	Escape window	High/low signal
Escape window	1	
High/low signal	\	1

**Table:**

	Modeling	Painting
Modeling	1	
Painting	\	1

**Table:**

	Material level	System level	Spiritual level
Material level	1		
System level	\	1	
Spiritual level	\	\	1

### B. Scoring questionnaires of correlation degree of design schemes

Table 12 shows the images of the design schemes and sample analysis chart of the escape window visual simulation, with the latter as the scoring basis of the escape window H_1_.

Table 13 shows the evaluation scales and meanings when scoring each index of the design schemes.

**Table 13:** Scheme evaluation scales and meanings.

No	Evaluation scales	Scoring range
1	Failed	[0, 59]
2	Barely passed	[60, 69]
3	Average	[70, 79]
4	Good	[80, 89]
5	Excellent	[90, 100]

Please score the evaluation indices corresponding to the three design schemes in the table below in accordance with the renderings and escape window virtual simulation analysis chart, in which the line of “high/low signal H_2_” can be ignored.

**Table:**

	Scheme 2	Scheme 3	Scheme 4
Relevant standards and technical conditions T_1_			
Train body structure T_2_			
Materials and techniques T_3_			
Escape window H_1_			
High/low signal H_2_	\	\	\
Modeling A_1_			
Painting A_2_			
Material level C_1_			
System level C_2_			
Spiritual level C_3_			

### C. Questionnaire of index scoring of the design schemes

**1. Explanation of the expert version of the subway train body design questionnaire**

- **Questionnaire content:** This is a questionnaire for evaluating the exterior design schemes of Guangzhou subway train. It requires you to score the three design schemes from several aspects (will take about 3–5 min).
- **Target group:** Subway experts who understand the body structure of subway trains and related technical standards.
- **Questionnaire reward:** An effective questionnaire will be paid 20 yuan, and the upper limit of the effective questionnaires is 10 copies (to ensure a smooth payment, please make sure to fill in your real name after the questionnaire is submitted).
- **Questionnaire link (10 × 3 = 30 copies in total):**
  1. Evaluation of exterior design schemes of Guangzhou subway train (feedback from 10 experts from CRRC Changchun Railway Vehicles Co., Ltd.): https://www.wjx.cn/report/179611253.aspx
  2. Evaluation of exterior design schemes of Guangzhou subway train (feedback from 10 experts from CRRC Qingdao Sifang Co., Ltd.): https://www.wjx.cn/report/180457747.aspx
  3. Evaluation of exterior design schemes of Guangzhou subway train (feedback from 10 experts from CRRC Tangshan Co., Ltd.): https://www.wjx.cn/report/180455773.aspx

**2. Explanation of the subway passenger version of the questionnaire**

- **Questionnaire content:** This is a questionnaire for evaluating the exterior design schemes of Guangzhou subway train. It requires you to score the three design schemes from the aspect of the subway train exterior (will take about 3–5 min).
- **Target group:** Subway train passengers who understand the regional culture of Guangzhou.
- **Questionnaire reward:** An effective questionnaire will be paid 5 yuan, and the upper limit of effective questionnaires is 40 copies (to ensure a smooth payment, please make sure to fill in your real name after the questionnaire is submitted).
- **Questionnaire link (40 × 8 = 320 copies in total):**
  1. Evaluation of exterior design schemes of Guangzhou subway train (feedback from passengers in group 1): https://www.wjx.cn/report/180439534.aspx
  2. Evaluation of exterior design schemes of Guangzhou subway train (feedback from passengers in group 2): https://www.wjx.cn/report/180445848.aspx
  3. Evaluation of exterior design schemes of Guangzhou subway train (feedback from passengers in group 3): https://www.wjx.cn/report/180460519.aspx
  4. Evaluation of exterior design schemes of Guangzhou subway train (feedback from passengers in group 4): https://www.wjx.cn/report/180454316.aspx
  5. Evaluation of exterior design schemes of Guangzhou subway train (feedback from passengers in group 5): https://www.wjx.cn/report/180985208.aspx
  6. Evaluation of exterior design schemes of Guangzhou subway train (feedback from passengers in group 6): https://www.wjx.cn/report/180980078.aspx
  7. Evaluation of exterior design schemes of Guangzhou subway train (feedback from passengers in group 7): https://www.wjx.cn/report/180993484.aspx
  8. Evaluation of exterior design schemes of Guangzhou subway train (feedback from passengers in group 8): https://www.wjx.cn/report/180988941.aspx
